# Study on the Use of Cooking Oil in Chinese Dishes

**DOI:** 10.3390/ijerph16183367

**Published:** 2019-09-12

**Authors:** Gangwei Pu, Mo Zheng, Shijun Lu, Jiazhang Huang

**Affiliations:** Institute of Food and Nutrition Development, Ministry of Agriculture and Rural Affairs, Beijing 100081, China

**Keywords:** cooking oil, Chinese cuisine, dining out, diet, nutrition

## Abstract

The purpose of this study was to research the amount of cooking oil used in mainstream Chinese cuisine, as well as the features of cooking oil used in different types of dishes. The results provide reference data for the assessment of edible oil intake for eating out. A total of 302 common Chinese dishes were chosen and prepared following standard procedures. The cooking oils used in these dishes were analyzed in terms of the cooking process, food material combination, types of primary food materials, type of cooking oil, and the purpose of the cooking oil. The results showed that the mean amount of cooking oil used per 100 g of food material was 8.1 g. There were no significant differences in the amount of cooking oil used in the eight major styles of Chinese cuisine. The average amount of cooking oil used in hot dishes (10.0 g) was higher than that used in cold dishes. The amount of cooking oil in pure meat, mixed meat-vegetable, and pure vegetable dishes was up to 10.9 g, 9.3 g, and 4.6 g, respectively. The findings of the present study could be used in future dietary surveys to determine the average oil consumption associated with different dishes. In addition, the results provide reference data for oil intake assessments in nutrition surveys or nutritional recipes.

## 1. Introduction

The level of cooking oil exposure in foods is closely related to the occurrence of chronic, non-infectious diseases [1,2,3]. Edible oil is an important source of essential fatty acids and vitamin E, but excessive oil intake greatly increases the risk developing obesity, coronary heart disease, and diabetes [4,5]. Current guidelines recommend that adults limit their oil intake to 25–30 g per day [6,7]. It is important to determine the typical amount of cooking oil used in frequently eaten dishes to determine whether balanced nutritional meal preparation is occurring. In recent years, it has become increasingly convenient to dine out [8,9], and a greater variety of dishes has emerged, leading to dramatic changes in the food consumption of Chinese residents. There are many styles of Chinese cuisine that are cooked in different ways and follow no fixed standard. Therefore, it is difficult to determine the amount of oil that Chinese people consume daily, particularly when food is purchased rather than prepared at home [10]. In addition, the three-day weighting method and food frequency questionnaires that are typically used in nutrition surveys [11,12] are less precise in estimating the amount of seasonings such as cooking oil. Thus, by researching the amount of cooking oil used in mainstream Chinese cuisine, as well as the features of cooking oil used in different types of dishes, this study aimed to provide reference data for oil intake assessments in nutrition surveys or nutritional recipes.

## 2. Materials and Methods

### 2.1. Sampling and Methods

Based on a review of the literature [13,14], combined with expert opinions from the China Cuisine Association, the dishes studied in this paper were chosen in accordance with the following principles: (1) They were prepared with a great diversity of food materials, (2) traditional cooking methods were used, and (3) hot and cold meat and vegetable dishes were incorporated. In total, 8 major styles of Chinese cuisine incorporating 302 mainstream dishes were selected [15]: 33 Sichuan, 33 Huaiyang, 69 home, 43 Shandong, 37 Halal [16], 22 northwest [17], 33 Xiange, and 32 Cantonese dishes.

We analyzed the cooking oil used in these dishes from the perspective of the cooking process, alongside the combination of food materials, the types of primary food materials, type of cooking oil, and purpose of the cooking oil’s use. Based on the cooking process, the dishes were divided into cold and hot dishes. The constituent ratio of cold and hot dishes varied for different styles of dishes. Based on the combination of food materials, the dishes were divided into pure vegetable, pure meat, and mixed meat-vegetable dishes. Based on the types of primary food materials, dishes were divided into vegetable, soy product, aquatic product, pork, beef and mutton, and poultry dishes. Based on the type of cooking oil, dishes were divided based on the use of one, two or three types of cooking oil. Based on the purpose of cooking oil use, dishes were divided by heating and processing, adding flavor, and seasoning. Cooking oil used for heating and processing refers to oil added as a heat transfer medium for sautéing, stir frying or deep frying, for example, soybean and peanut oils. Sesame oil was the oil most commonly used for adding flavor to dishes. Oils for seasoning usually referred to oils mixed with other condiments and used to give a new taste to the dishes, for example, pepper essential and chili oils.

### 2.2. Dish Preparation and Quality Control

The food material selection criteria, the combination of food materials, and the cooking methods for each dish were based on the recommendations of national culinary masters. The amounts of primary and secondary food materials and the sauces used for each dish were calculated by the investigator. Professional chefs were hired to cook these dishes on site, and all food materials used for cooking were purchased from the market. To reduce the memory bias caused by culinary masters recalling the doses of sauces, 5 judges evaluated the taste of the dishes in terms of saltiness and greasiness after each dish was cooked and prepared. The dose of sauce used for each dish was adjusted based on this evaluation.

### 2.3. Calculation of Cooking Oil Dosage

The amount of cooking oil used for each dish was determined as the amount per 100 g of the edible parts of the primary and secondary food materials (excluding the liquid added during the cooking process, such as water or broth): The amount of cooking oil (g/100 g) = actual amount of cooking oil used (g) ÷ ∑ raw weight of the edible parts of the primary and secondary food materials (g) × 100.

### 2.4. Statistical Analysis

Statistical analyses were performed using IBM SPSS Statistics 24.0 software (IBM, Armonk, New York, USA). Data are expressed as the mean (standard deviation) and median (interquartile range). If the data did not obey a normal distribution, then an intergroup comparison was performed using the Mann-Whitney test. Multiple comparisons were performed using the Kruskal-Wallis rank sum test. *p* < 0.05 indicated a significant difference.

## 3. Results

### 3.1. Dishes Divided Based on the Cooking Process

The mean amount of cooking oil used per 100 g of raw food materials in the dishes was 8.1 g, and there was no significant difference in amount of oil used between the different styles of dishes. However, a greater amount of cooking oil was used in the hot (10.0 g per 100 g of raw food materials) and oil-cooked hot (0.4~39.4 g) dishes (mean, 12.0 g; median, 10.2 g) than in the cold (4.2 g) and oil-cooked cold (0.7~17.1 g) dishes (mean, 5.6 g; median, 4.5 g) (*p* < 0.05; Table 1).

### 3.2. Dishes Divided by Food Material Combination

There were significant differences in the amount of cooking oil used in the pure vegetable, pure meat, and mixed meat-vegetable dishes (*p* < 0.05; Table 2). The mean amount of cooking oil used per 100 g of raw food materials was 4.6 g in the pure vegetable dishes, 0.9~16.7 g (mean, 5.6 g; median, 4.7 g) in the oil-cooked pure vegetable dishes, 10.9 g in the pure meat dishes, 0.4~35.3 g (mean, 14.2 g; median, 12.8 g) in the oil-cooked pure meat dishes, 9.3 g in the mixed meat-vegetable dishes, and 0.7~39.4 g (mean, 11.0 g; median, 9.3 g) in the oil-cooked mixed meat-vegetable dishes.

### 3.3. Dishes Divided by the Type of Primary Food Materials 

**Figure 1:**
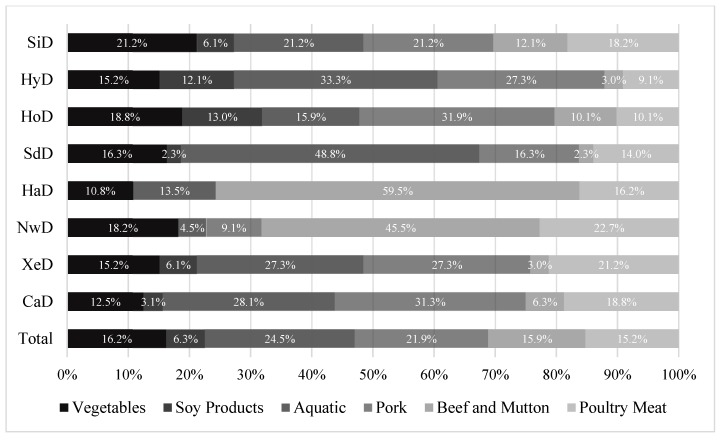
Constituent ratios of different types of dishes for each style.

The constituent ratio of the different primary food materials was more balanced in the Sichuan dishes. Aquatic product dishes accounted for one-third of Huaiyang dishes, pork dishes accounted for > 30% of home and Cantonese dishes, aquatic product dishes accounted for nearly one-half of the Shandong dishes, and beef and mutton dishes accounted for > 40% of Halal and northwest dishes. Of all styles of dishes, meat dishes accounted for > 50% (Figure 1).

Of all dishes cooked with oil, the amount of cooking oil was the lowest in dishes with soy products as the primary food materials (median, 4.6 g) and highest in dishes with aquatic products and pork as their primary food materials (median, 11.8 g). The amount of cooking oil used in dishes with vegetables and soy products as their primary food materials was lower than in dishes primarily made of aquatic products, pork, beef and mutton, and poultry (*p* < 0.05; Table 3).

### 3.4. Type of Cooking Oil Used in the Dishes

The amount of cooking oil (*p* < 0.05; Table 4) varied depending on the number of oils used: 18.2% of dishes were cooked with no oil; 65.9% used one type of oil and used a mean oil quantity of 9.9 g per 100 g of raw food materials; 14.9% used two types of oil (mean oil quantity: 13.1 g); and 1.0% used three types of oil (mean oil quantity: 25.9 g).

### 3.5. Purpose of Cooking Oil Use

The amount of cooking oil (*p* < 0.05; Table 5) used was generally 0.8~39.4 g per 100 g of raw food materials (mean, 11.9 g; median, 10.4 g) for preliminary heating and processing, 0.2~14.0 g (mean, 2.6 g; median, 1.6 g) to add flavor, and 0.9~11.7 g (mean, 4.9 g; median, 3.9 g) for seasoning.

## 4. Discussion and Limitations

The present study, one of the few large-sample studies based on self-measured data in this field, determined the amount and features of cooking oil use in 302 mainstream dishes belonging to different styles of food in the Chinese catering market. The cooking methods and food material selections all conformed to the standards of each major dish style. The research findings further complement the basic database on the nutritional composition of Chinese cuisine and also provide data to help with the development of dietary surveys and healthy, nutritional recipes.

The mean amount of cooking oil used per 100 g of raw food materials in Chinese cuisine was 8.1 g. According to the Chinese Residents’ Nutrition and Health Survey 2012 [18], the daily food intake of Chinese residents is 419.7 g, suggesting that oil consumption per capita was of 34.0 g (419.7 g × 8.1 g/100 g) in this study. This amount is lower than the value published by the Comprehensive Report, Chinese Residents’ Nutrition and Health Survey 2010–2013 (37.1 g). Chen et al. [19] performed a survey on 34 group dinner dishes and reported that the amount of cooking oil used per 100 g of raw food materials was 5.9 g. This is lower than our reported result, possibly because the group dinner industry has been developing rapidly in recent years, and the trend towards more nutritional and standard dishes in group dinners [20] has led to blander dishes compared to those cooked at restaurants. Accordingly, similar to our results, Cao et al. [21] reported that the mean amount of cooking oil used per 100 g of raw food materials in dishes cooked at restaurants in Beijing, Shanghai, and Guangdong is 8.1 g. Wang et al. [22] reported that the amount of cooking oil consumed per standard person per meal at a restaurant is 28 g in Beijing, which is equivalent to 7.7 g per 100 g of raw food materials. This finding is similar to our home dish (HoD) result, which makes sense because there are many restaurants that serve HoD in Beijing. In addition, globally and within China, there has been a movement towards reducing the amount of oil and salt used in cooking, which has led to a positive boost to edible oil reduction actions in major cities such as Beijing. This is also the main reason the results of this study are slightly lower than those of Wang et al.

In the present study, the amount of cooking oil used was found to be significantly lower in cold dishes than in hot dishes. This is because cooking methods are easier for cold dishes, and oils are generally used for adding flavor and seasoning. In contrast, the cooking methods for hot dishes are more diverse, and because when oil is added for heating and processing, it is added in a greater amount. The amount of cooking oil used in pure vegetable dishes was found to be much lower than that used in pure meat dishes because the vegetables are usually sautéed over a large fire. In contrast, meat is cooked via stir-frying and deep-frying processes, which consumes more oil. The higher consumption of oil in meat dishes may also be related to the following factors: Firstly, some water will flow out during meat cooking, and high temperatures facilitate the volatilization of oil. Secondly, meat is usually cut into small blocks, so both the inner and outer sides of the meat will be cooked thoroughly. As the surface area of the meat increases, the meat will absorb more cooking oil. There is a saying that “all dishes can be cooked well as long as there is much oil.” Finally, adding a sufficient amount of cooking oil enhances the visual appearance of a dish.

We found no significant difference in the amount of cooking oil used for different styles of dishes, because many diverse food materials are used in the dishes of each style. Although there was a large difference in the amount of cooking oil used for different types of food materials, the overall differences were small. Only one type of oil was used in approximately two-thirds of the dishes in the present study, and approximately one-half of the dishes were cooked with soybean oil. This may be affected by usage habits and convenience.

The present study has certain limitations. First, all data on oil consumption were collected by weighing, and the amount of oil lost by volatilization during cooking was not calculated. Second, the number of dishes included was limited. Only mainstream dishes on the market were studied, and the results do not represent all Chinese dishes. Future research will attempt to overcome the above limitations.

## 5. Conclusions

This study investigated the amount of cooking oil used in mainstream Chinese cuisine as well as the features of cooking oil used in different types of dishes. No significant differences were measured in the amount of cooking oil used in different styles of dishes. However, on the whole, more cooking oil was used in hot dishes than in cold dishes, and more oil was used in meat dishes than in pure vegetable dishes. The findings of the present study can be used in future dietary surveys to determine the average oil consumption associated with different dishes.

## Figures and Tables

**Table 1:** Amount of cooking oil used per 100 g of raw food materials in cold and hot dishes.

Type	CD ^(1)^				HD^(2)^				CD/HD	M^W^
	N(n)	M (SD)	M^O^(SD^O^)	Median^O^ (IQR^O^)	N(n)	M(SD)	M(SD)	Median^O^ (IQR^O^)		
SiD	8 (8)	7.5 (5.0)	7.5 (5.0)	7.5 (6.0)	25 (22)	11.8 (10.0)	13.4 (9.5)	12.1 (10.7)	8/8	9.7
HyD	7 (5)	7.8 (6.5)	11.0 (4.5)	13.8 (4.4)	26 (23)	10.6 (10.1)	12.0 (10.0)	8.1 (8.5)	8/12	9.4
HoD	5 (4)	5.8 (5.2)	7.3 (4.7)	6.0 (6.1)	64 (57)	9.1 (7.7)	10.3 (7.4)	10.0 (9.0)	6/8	7.7
SdD	16 (11)	1.6 (1.8)	2.3 (1.8)	1.5 (2.4)	27 (24)	10.7 (8.7)	12.0 (8.2)	11.4 (12.1)	6/10	7.3
HaD	8 (5)	2.0 (2.4)	3.1 (2.4)	2.3 (0.5)	29 (18)	6.9 (7.1)	11.1 (5.8)	11.5 (7.0)	6/10	5.1
NwD	4 (3)	3.5 (2.5)	4.7 (1.3)	4.5 (2.5)	18 (13)	10.5 (16.5)	14.5 (18.0)	9.7 (12.8)	8/8	7.0
XeD	6 (4)	3.0 (2.6)	4.5 (1.5)	5.0 (2.1)	27 (23)	11.0 (12.9)	12.9 (13.1)	9.3 (13.9)	4/10	8.7
CaD	6 (5)	6.0 (5.1)	7.3 (4.7)	8.1 (7.1)	26 (22)	11.1 (10.0)	13.1 (9.5)	11.7 (12.8)	4/10	9.6
Total	60 (44)	4.2 (4.5)	5.6 (4.4)	4.5 (6.4)	242(202)	10.0 (10.0)	12.0 (9.8)	10.2 (11.6)	-	8.1

SiD = Sichuan dish; HyD = Huaiyang dish; HoD = Home dish; SdD = Shandong dish; HaD = Halal dish; NwD = Northwest dish; XeD = Xiange dish; CaD = Cantonese dish; CD = cold dish; HD = hot dish; N = total number of dishes; n = number of oil-cooked dishes; M = mean; SD = standard deviation; IQR = interquartile range; M^O^ = mean amount of cooking oil in oil-cooked dishes; SD^O^ = standard deviation of the amount of cooking oil in oil-cooked dishes; Median^O^ = the median of amount of cooking oil in oil-cooked dishes; IQR^O^ = the interquartile range of the amount of cooking oil in the oil-cooked dishes; CD/HD = the number of cold/hot dishes (there were 10 people at each dinner table, and the number of cold and hot dishes and the types of dishes served were determined by the national culinary masters); M^W^ = Weighted Mean; *p* < 0.05 based on a comparison of (1) and (2).

**Table 2:** Amounts of cooking oil used per 100 g of raw food materials in the pure vegetable, pure meat, and mixed meat-vegetable dishes.

Type	Pure Vegetable^(1)^				Pure Meat ^(^^2)^				Mixed Meat-Vegetable^(3)^			
	N (n)	M (SD)	M^O^ (SD^O^)	Median^O^ (IQR^O^)	N(n)	M(SD)	M^O^ (SD^O^)	Median^O^ (IQR^O^)	N (n)	M (SD)	M^O^ (SD^O^)	Median^O^ (IQR^O^)
SiD	6 (5)	4.1 (3.4)	4.9 (3.1)	5.1 (2.7)	8 (8)	13.7 (9.1)	13.7 (9.1)	11.6 (11.8)	19 (17)	11.6 (9.7)	13.0 (9.3)	11.3 (10.0)
HyD	8 (8)	7.2 (4.4)	7.2 (4.4)	6.1 (4.4)	10 (8)	12.9 (9.1)	16.2 (6.9)	14.0 (3.6)	15 (12)	9.5 (11.4)	11.9 (11.6)	7.8 (4.4)
HoD	19 (17)	5.1 (4.1)	5.7 (3.9)	4.9 (4.1)	15 (12)	12.6 (10.6)	15.8 (9.4)	13.1 (6.9)	35 (32)	9.4 (6.7)	10.3 (6.3)	10.8 (8.6)
SdD	6 (4)	4.3 (5.1)	6.5 (4.9)	6.2 (6.8)	11 (8)	12.9 (11.4)	17.7 (9.3)	18.9 (16.9)	26 (23)	5.6 (6.1)	6.4 (6.2)	3.5 (9.4)
HaD	5 (2)	0.9 (1.2)	2.2 (0.4)	2.2 (0.5)	11 (7)	6.7 (7.8)	10.6 (7.3)	12.5 (16.3)	21 (14)	6.5 (6.6)	9.8 (5.6)	9.6 (8.1)
NwD	4 (4)	6.7 (4.2)	6.7 (4.2)	5.2 (5.4)	8 (4)	6.2 (10.6)	12.4 (12.6)	11.4 (21.5)	10 (8)	12.6 (20.4)	15.7 (21.9)	8.6 (8.6)
XeD	6 (4)	2.8 (2.4)	4.2 (1.5)	4.5 (1.8)	11 (9)	14.7 (18.1)	17.9 (18.5)	10.5 (13.6)	16 (14)	8.6 (7.4)	9.8 (7.1)	7.9 (11.3)
CaD	4 (3)	3.1 (2.2)	4.2 (0.8)	4.4 (1.5)	11 (9)	6.3 (5.4)	7.7 (5.0)	8.1 (7.1)	17 (15)	14.2 (10.5)	16.1 (9.7)	16.4 (11.9)
Total	58 (47)	4.6 (4.0)	5.6 (3.7)	4.7 (3.4)	85 (65)	10.9 (11.0)	14.2 (10.5)	12.8 (11.4)	159 (135)	9.3 (9.5)	11.0 (9.4)	9.3 (11.4)

N = the total number of cold and hot dishes; n = the number of oil-cooked dishes. *p* < 0.05 comparing (1), (2), and (3) in the oil-cooked dishes.

**Table 3:** Amounts of cooking oil used per 100 g of raw food materials in dishes with different types of primary food materials.

Item	Food Category	Lower Quartile^O^	Median^O^	Upper Quartile^O^	χ^2^
Amount of cooking oil	Vegetables	3.2	4.8^(1)^	7.5	26.236 *
	Soy Products	2.6	4.6^(1)^	7.0	
	Aquatic	4.5	11.8^(2)^	18.3	
	Pork	5.8	11.8^(2)^	17.4	
	Beef and Mutton	5.5	8.0^(2)^	16.1	
	Poultry Meat	6.1	8.4^(2)^	12.3	

* *p* < 0.05 based on a comparison of (1) and (2).

**Table 4:** Amount of cooking oil used per 100 g of raw food materials in cold and hot dishes.

Type	One Type of Oil^(1)^			Two Types of Oil^(2)^			Three Types of Oil^(3)^	
	n	M^O^(SD^O^)	Median^O^ (IQR^O^)	n	M^O^ (SD^O^)	Median^O^ (IQR^O^)	n	M^O^
SiD	18	10.9 (7.3)	9.8 (8.3)	12	13.2 (11.1)	8.6 (11.8)	0	-
HyD	26	11.8 (9.5)	8.8 (8.1)	2	11.0 (3.2)	11.0 (4.5)	0	-
HoD	57	9.5 (6.5)	8.5 (9.3)	3	17.3 (16.1)	12.0 (30.8)	1	19.7
SdD	27	8.5 (9.0)	5.3 (15.3)	8	10.5 (5.2)	10.8 (9.2)	0	-
HaD	20	8.3 (5.7)	7.5 (9.3)	3	16.5 (5.4)	14.0 (9.9)	0	-
NwD	14	9.2 (7.4)	6.8 (9.3)	1	4.5	4.5	1	39.4
XeD	15	10.0 (15.1)	5.6 (7.9)	11	13.3 (8.5)	9.4 (15.0)	1	18.6
CaD	22	11.5 (9.6)	10.8 (12.4)	5	14.3 (6.1)	14.4 (9.0)	0	-
Total	199	9.9 (8.5)	7.5 (10.0)	45	13.1 (8.6)	11.8 (11.3)	3	25.9

(1), (2), and (3) represent the number of types of cooking oil used in each style of dish. Dishes cooked with one type of oil commonly use soybean oil/salad oil/blend oil in hot dishes and sesame oil in cold dishes. Dishes cooked with two types of oil use a combination of soybean oil and sesame oil and soy oil and chili oil and are usually hot dishes cooked with a more complex process. Dishes cooked with three types of oil commonly use the combination of soy, chili, and sesame oils.

**Table 5:** Amount of cooking oil used per 100 g of raw food materials in dishes for different purposes.

Type	Preliminary Heating and Processing			Adding Flavor			Seasoning		
	n	M^O^ (SD^O^)	Median^O^ (IQR^O^)	n	M^O^ (SD^O^)	Median^O^ (IQR^O^)	n	M^O^ (SD^O^)	Median^O^ (IQR^O^)
SiD	21	13.4 (9.5)	12.8 (9.1)	12	1.9 (2.7)	1.1 (1.3)	9	5.6 (3.2)	5.7 (6.1)
HyD	26	11.5 (9.4)	8.4 (7.8)	3	6.2 (6.7)	3.7 (12.6)	0	-	-
HoD	50	10.8 (7.1)	11.5 (8.3)	13	4.1 (3.5)	3.4 (3.3)	3	6.2 (4.8)	3.6 (8.6)
SdD	24	11.8 (8.1)	11.1 (11.7)	15	1.7 (1.2)	1.3 (1.0)	2	2.4 (0.1)	2.4 (0.1)
HaD	18	10.9 (5.5)	10.8 (7.0)	6	1.8 (1.1)	1.8 (1.2)	2	3.5 (1.7)	3.5 (2.3)
NwD	12	15.1 (16.7)	9.8 (13.9)	4	3.1 (2.3)	2.6 (3.5)	2	4.4 (2.2)	4.4 (3.2)
XeD	24	11.5 (12.8)	7.9 (12.8)	8	2.3 (1.6)	1.7 (2.8)	4	4.9 (2.9)	5.6 (3.5)
CaD	25	12.9 (8.9)	11.2 (11.7)	5	1.6 (1.0)	1.2 (1.4)	1	3.6 (-)	3.6 (-)
Total	200	11.9 (9.4)	10.4 (10.6)	66	2.6 (2.7)	1.6 (2.3)	23	4.9 (3.0)	3.9 (4.6)
