# Erratum: Pu, G., *et al.* Study on the Use of Cooking Oil in Chinese Dishes. *Int. J. Environ. Res. Public Health* 2019, *16*, 3367

**DOI:** 10.3390/ijerph17061932

**Published:** 2020-03-16

**Authors:** Gangwei Pu, Mo Zheng, Shijun Lu, Jiazhang Huang

**Affiliations:** Institute of Food and Nutrition Development, Ministry of Agriculture and Rural Aairs, Beijing 100081, China; pugangwei@163.com (G.P.); zhengmo@caas.cn (M.Z.)

In the original version of our article [1], insufficient acknowledgement was given for the origins of some of the dish samples used. We apologize for the original error. To correct this oversight, Guangyan Cheng has been added as an author, and the acknowledgements have been altered to more appropriately recognize support and funding.

The original and corrected author list, author contributions and acknowledgements are provided below:

## 1. Original Particular

**Author List:** Gangwei Pu, Mo Zheng, Shijun Lu (Corresponding author), and Jiazhang Huang (Corresponding author).

**Author Contributions**: Conceptualization, S.L.; formal analysis, M.Z.; writing—original draft preparation, G.P.; writing—review and editing, J.H.

**Acknowledgments**: Thanks to the Chinese academy of agricultural sciences science and technology innovation project for funding this survey. Thanks to Lin Tong, Tong Zhang, Weijun Yao, Jianzhong Wang, Bogeng Deng, Yunlong Zhu, Qiang Liu, Shudong Guan, Zuming Chen, and Gang Cheng, for their efforts and hard work in making dishes. Thanks to Xin Meng Tai He hotel management (Beijing) Co., Ltd. for the processing of common dishes to give strong support. Thanks to Tingting Wang for her help in the manuscript’s statistical methods. 

## 2. Corrected Particular

**Author List:** Gangwei Pu (First author), Shijun Lu (First author), Mo Zheng, Jiazhang Huang (Corresponding author), Guangyan Cheng (Corresponding author).

**Author Contributions**: Conceptualization, S.L.; formal analysis, M.Z.; writing—original draft preparation, G.P.; writing—review and editing, J.H.; Data curation, G.C.

**Acknowledgments**: Thanks to the Chinese academy of agricultural sciences science and technology innovation project for funding this survey. Thanks to our team members Lin Zhou, Zhenni Yang and Zhenchuang Tang for their help in all aspects of the project. Thanks to Lin Tong, Tong Zhang, Weijun Yao, Jianzhong Wang, Bogeng Deng, Yunlong Zhu, Qiang Liu, Shudong Guan, Zuming Chen, and Gang Cheng, for their efforts and hard work in making dishes. Thanks to Xin Meng Tai He hotel management (Beijing) Co., Ltd. for the processing of common dishes to give strong support. Thanks to Tingting Wang for her help with the manuscript’s statistical methods.

These changes do not influence the results, discussion, or conclusions. The authors would like to apologize for any inconvenience caused to the readers by this error.
